# Anterior chest wall tuberculous abscess: a case report

**DOI:** 10.1186/1752-1947-1-152

**Published:** 2007-11-26

**Authors:** Theodossis S Papavramidis, Vassilis N Papadopoulos, Antonis Michalopoulos, Daniel Paramythiotis, Stamatia Potsi, Georgia Raptou, Anna Kalogera-Foutzila, Nick Harlaftis

**Affiliations:** 1 1st Propedeutic Surgical Clinic, Aristotle's University of Thessaloniki, A.H.E.P.A. University Hospital, Thessaloniki, Greece; 2 Department of Radiology, Aristotle's University of Thessaloniki, A.H.E.P.A. University Hospital, Thessaloniki, Greece; 3 Department of Pathology, Aristotle's University of Thessaloniki, School of Medicine, Greece

## Abstract

The granulomatous inflammation of tuberculosis usually involves the lungs and the hilar lymph nodes. Musculoskeletal tuberculosis (TB) occurs in 1–3% of patients with TB, while TB of the chest wall constitutes 1% to 5% of all cases of musculoskeletal TB. Furthermore, nowadays it is rarer to find extrapulmonary TB in immunocompetent rather that non-immunocompetent patients. The present case reports a fifty-six-year-old immunocompetent man with an anterior chest wall tuberculous abscess. The rarity of the present case relates both to the localization of the tuberculous abscess, and to the fact that the patient was immunocompetent. The diagnosis of musculoskeletal tuberculous infection remains a challenge for clinicians and requires a high index of suspicion. The combination of indolent onset of symptoms, positive tuberculin skin test, and compatible radiographic findings, strongly suggests the diagnosis. TB, however, must be confirmed by positive culture or histologic proof. Prompt diagnosis and treatment are important to prevent serious bone and joint destruction.

## Introduction

Musculoskeletal tuberculosis (TB) occurs in 1–3% of patients with TB [1,2], while TB of the chest wall constitutes 1% to 5% of all cases of musculoskeletal TB [3]. Tuberculous tendon and muscle infection may result from direct inoculation or hematogenous dissemination from a primary focus such as the lung [4].

This case report is interesting because it reports a case of chest wall TB in an immunocompetent patient.

## Case presentation

**Figure 1:**
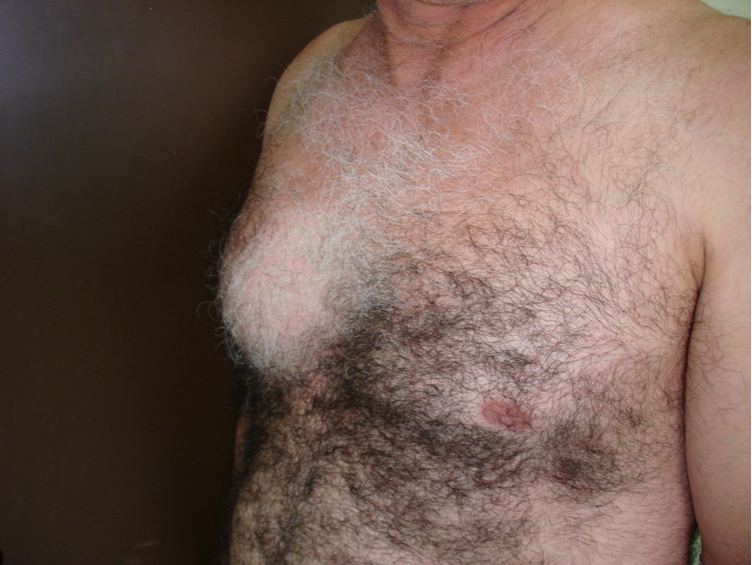
3/4 of a left profile of the patient showing the swelling of the right anterior thoracic wall.

**Figure 2:**
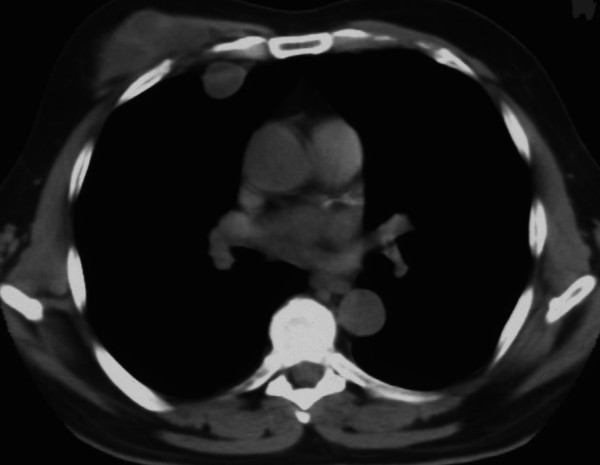
CT axial scans of the thorax showing an intramuscular hypodense tumor with peripheral enhancement which adheres to the body of the right 4^th ^rib. Smaller soft tissue attenuation tumor diameter 2 cm

A fifty-six-year-old man who immigrated to Greece from Georgia (former USSR) came to the emergency department for evaluation of a gradually enlarging soft tissue mass in the right anterior thoracic wall. The mass had been present for fifteen years. During the last three months, it had grown more rapidly than before and the patient suffered from local pain. The medical history of this patient was unremarkable. He had no pulmonary symptoms, such as a productive cough, or other symptoms. Examination revealed that the patient was afebrile and well nourished. The findings on clinical examination were unremarkable, except for a large, firm, mobile (but with small movement amplitude), and tender soft tissue mass in the right anterior thoracic wall (Fig. 1). The white blood cell count was normal. All biochemical examinations as well as erythrocyte sedimentation rate were within normal ranges. C-reactive protein was 3.82 mg/dl (normal 0.–0.8 mg/dl). A tuberculin test was not performed. HIV test was performed and was negative. A plain radiograph of the chest revealed normal findings. CT showed two cystic formations: a large cystic formation between the pectoralis major and minor muscles adjacent to the costal cartilage and a small one in the thoracic cavity, at the same height as the large one. As measured with CT imaging, this large mass was approximately 11 cm in length, 7 cm in width, and 4.5 cm in anteroposterior diameter, while the small one had a diameter of about 2 cm (Fig. 2). The overlying skin appeared normal, with no wounds, scars, rash, or sinuses. At the time of operation, the subcutaneous tissue was found to be oedematous. Deeper dissection, within the muscle layers, revealed a large abscess. About 300 ml of turbid, serous fluid poured out of the cystic formation. As there was no evidence of any neoplasm, the soft tissues were then debrided and specimens were send for histological and microbiological analysis. The tissue fragments submitted for histologic examination consisted of fibroconnective tissue with necrotizing granulomatous inflammation. There were large areas of necrosis, which were lined by epithelioid histiocytes, Langhans type giant cells and fibroblasts. *Mycobacterium tuberculosis *grew on culture of a specimen of the tissue and this was further confirmed with PCR Amplicor (Roche). A drain was placed in the wound and a large dressing was applied over the right thoracic wall. The patient is currently receiving daily antituberculous chemotherapy, consisting of isoniazid (300 mg), rifampin (600 mg), pyrazinamide (2 g), and pyridoxine (50 mg). The postoperative course has been uneventful and a postoperative CT showed no recurrence of the drained tuberculoma.

## Discussion

Tuberculous abscesses of the chest wall can involve the sternum, costochondral junctions, rib shafts, costovertebral joints and the vertebrae. They are most frequently found at the margins of the sternum and along the rib shafts [5]. The parasternal region, costovertebral junction, and vertebra are involved less frequently [3]. Chung et al. have previously reported CT scanning to be a useful tool for investigation in the diagnosis of a tuberculous retromammary abscess and also in determining the extent of an abscess [6]. Our case involved a tuberculous abscess which extended between the pectoralis muscles with involvement and erosion of the costal cartilage.

Tuberculous muscle infection may result from direct inoculation or hematogenous dissemination from a primary focus such as the lung [4]. The onset of symptoms of tuberculous tenosynovitis is typically gradual, with slow progressive swelling, ensuing pain, and decreased range of motion [7-9]. In our case the patient had had the tuberculoma for at least 15 years without symptoms, but had then experienced a rapid augmentation of its volume over the last 3 months.

Tuberculous infections have been increasing in incidence during the last decades for a variety of reasons, including increasing numbers of patients with immunity-depressive diseases, drug resistance, aging population, and health care worker exposure [10]. None of the above was relevant in this case of TB however our patient was an immigrant from an area with a high prevalence of TB.

## Conclusion

The granulomatous inflammation of tuberculosis usually involves the lungs and the hilar lymph nodes, and anterior chest wall involvement is very rare. Furthermore, nowadays it is rarer to find extrapulmonary TB involving immunocompetent patients than non-immunocompetent. The diagnosis of musculoskeletal tuberculous infection remains a challenge for clinicians and requires a high index of suspicion. The combination of indolent onset of symptoms, positive tuberculin skin test, and compatible radiographic findings, strongly suggests the diagnosis. TB treatment is often started immediately after the appropriate microbiological and histological samples have been obtained if the clinical suspicion is high. TB, however, must be confirmed by positive culture or histologic proof. Prompt diagnosis and treatment are important to prevent serious bone and joint destruction.

## Competing interests

The author(s) declare that they have no competing interests.

## Authors' contributions

Papavramidis T.S. 1) Received the patient to the emergency department and participated to the operation. Treating doctor. 2) Involved in drafting the manuscript and revising it critically for important intellectual content. 3) Have given final approval of the version to be published.

Papadopoulos V.N. 1) Main surgeon. 2) Have been involved in revising the draft critically for important intellectual content. 3) Have given final approval of the version to be published.

Michalopoulos A. 1) Auxiliary surgeon. 2) Have been involved in revising the draft critically for important intellectual content. 3) Have given final approval of the version to be published.

Paramythiotis D. 1) Received the patient to the emergency department and consulted on the pharmaceutical treatment. 2) Have been involved in drafting the manuscript. 3) Has given final approval of the version to be published.

Potsi S. 1) Performed all imaging studying. 2) Has been involved in drafting the manuscript. 3) Has given final approval of the version to be published.

Raptou G. 1) Performed all pathologic evaluation. 2) Has been involved in drafting the manuscript. 3) Has given final approval of the version to be published.

Kalogera-Foutzila A. 1) Evaluated the imaging studies. 2) Has been involved in revising the draft critically for important intellectual content. 3) Has given final approval of the version to be published.

Harlaftis N. 1) Strategic planning for the treatment of the patient. 2) Has been involved in revising the draft critically for important intellectual content. 3) Has given final approval of the version to be published.

## Consent

Written informed consent was obtained from the patient for publication of this case report and any accompanying images. A copy of the written consent is available for review by the Editor-in-Chief of this journal.
